# An Effect of Molecular Motion on Carrier Formation in a Poly(3-hexylthiophene) Film

**DOI:** 10.1038/srep08436

**Published:** 2015-02-13

**Authors:** Yudai Ogata, Daisuke Kawaguchi, Keiji Tanaka

**Affiliations:** 1 Department of Applied Chemistry, Kyushu University, Fukuoka 819-0395, Japan; 2 Education Center for Global Leaders in Molecular Systems for Devices, Kyushu University, Fukuoka 819-0395, Japan; 3 International Institute for Carbon-Neutral Energy Research (WPI-I2CNER), Kyushu University, Fukuoka 819-0395, Japan

## Abstract

Free carriers, polarons (P), in conjugated polymers play a key role in the performance of optoelectronic devices. Here, we present solid evidence that P can be predominantly generated from polaron pairs (PP) in a poly(3-hexylthiophene) (P3HT) film under zero electric field. P formation from PP strongly depends on temperature. The temperature dependence of P starts to change around 300 K. P3HT exhibits a thermal molecular motion named the α_1_ relaxation process, in which the twisting motion of thiophene rings is released, in this temperature region. Thus, it can be claimed that the twisting motion of P3HT thiophene rings is one of the determining factors of the photodynamics of P in P3HT films. This finding holds true for poly(thiophene)s with different alkyl lengths and should be considered in the design and construction of highly-functionalized organic devices based on poly(thiophene)s.

Conjugated polymers have been widely studied because of their recognized potential in optoelectronic devices such as solar cells^1^,^2^,^3^, field-effect transistors^4^,^5^, and light-emitting diodes^6^,^7^. These materials possess a high absorption coefficient, are lightweight, have excellent mechanical flexibility and can be introduced with various functionalities. In particular, regioregulated poly(3-hexylthiophene) (P3HT), in which the hexyl side chain is attached to the third position of a thiophene ring in a head-to-tail regioregularity, has received considerable attention due to its excellent optoelectronic properties and solubility in organic solvents^8^,^9^,^10^.

In a solar cell, the P3HT component converts the incident light to charged carriers. Ideally, when P3HT molecules absorb photons, most of the incident photons can be converted to holes and electrons in principle, meaning that the internal quantum efficiency is up to ca. 80%^11^,^12^,^13^. If the molecular structure of the donor is optimized, the internal quantum efficiency can be 100%^14^. However, the power conversion efficiency is only around 6%^14^. To achieve a higher conversion efficiency, we still need a better understanding of the carrier formation mechanism beyond photoexcitation.

To address such concern, it is important to understand the carrier formation not only for polymer solar cells but also for the corresponding donor polymers. Here we focused on the carrier generation in the neat P3HT as a model donor polymer. Singlet excitons (**S**) and polaron pairs (**PP**), which are electron-hole pairs bound by Coulomb interaction, are generated in the initial stage of the photoexcitation. Free carriers, so-called polarons (**P**), are subsequently formed. Thus, to improve further the device performance, it is necessary to understand the fundamental mechanism that forms the **P** from other excited species. The formation dynamics of **P** after photoexcitation has been examined theoretically and experimentally^15^,^16^,^17^,^18^. A hot-exciton dissociation model has been proposed to explain the formation of **P** on a time scale of <100 fs in the high excitation energy^15^. This was experimentally confirmed by terahertz time domain spectroscopy^16^. In another study by Ohkita, Ito and co-workers, they have found that **P** can be formed from **S** in a P3HT film based on femtosecond transient absorption spectroscopy^17^. Also, **PP** can be dissociated into **P** under a sufficient electric field to overcome their Coulombic binding energy^18^. However, many researchers believe that **PP** cannot be efficiently dissociated into **P** without applying an electric field.

The conjugation length in a semiconducting polymer film generally depends on its preparation^19^,^20^. This is simply because the aggregation states of the polymer are affected by the preparation method. Thus, it is anticipated that the conjugation length is also a function of temperature, which activates the thermal molecular motion of the polymer. Actually, using solid-state ^13^C nuclear magnetic resonance (NMR)^21^ and dielectric relaxation spectroscopy (DRS)^22^, the relationship between the molecular motion and the conjugation length for P3HT has been discussed. A common conclusion drawn from these measurements is that the conjugation length is affected once the twisting motion of the thiophene rings is activated. However, it remains unclear how the change in the conjugation length induced by thermal molecular motion impacts the carrier formation in conjugated polymers.

The objective of this study is to attain a better understanding of this issue. The formation dynamics of **P** in P3HT films were examined as a function of temperature. By doing so, we have found out that **P** can be directly generated from **PP** without applying an electric field. Dynamic mechanical analysis (DMA) was also applied to the P3HT films so that the thermal molecular motion of P3HT in the film could be directly discussed. Combining these results, we come to the conclusion that the photodynamics of P3HT is strongly controlled by the chain dynamics, especially, the twisting motion.

A femtosecond transient absorption spectrum shows excited species generated after the photoexcitation. It has been established that **S**, **PP** and **P** are formed from hot-excitons within 100 fs and that their amounts depend on the power of the pump laser^15^,^17^. If the power of the pump laser is low, **P** is not formed while **PP** is produced within 100 fs^15^,^17^. In our experiment, the intensity of the pump laser was optimized to be low enough in order not to form **P** directly from hot-excitons. Figure 1 shows transient absorption spectra for a P3HT film at 300 K in the time range from 0 to 100 ps. The excitation was made by a light pulse with a wavelength of 400 nm. Panels (a) and (b) correspond to the data in visible and near infrared (NIR) regions, respectively. The time 0 is defined as the stage at which the bleaching intensity from the 0-2 transition around 520 nm is maximized for visible region. In this definition, the optical density (Δ*OD*) for **PP** around 650 nm also shows the maximum at *t* = 0. The time 0 also corresponds to the stage at which Δ*OD* of **S** shows the maximum for NIR region. This means that the conversion from hot-excitons to **S** and/or **PP** is completed within an infinitely short time. Hence, an effect of hot-excitons on other excited species could be neglected at *t* > 0 based from our analysis^17^. On the other hand, a positive peak arising from **P,** which should be observed around 1050 nm^17^, appeared approximately 1 ps later after the excitation. *ΔOD*(*t*) from **S** and **P** was extracted by a curve fitting method using Lorentzian function (Figures S1 and S2). The detail analysis of a transient spectrum is described in the supplementary information.

We analyze the data with coupled differential equations for the time dependence of concentration for **S**, **PP** and **P** taking all the possible relations among them into account. The merit of this method is that the transition of a transient species is evaluated not only on the basis of the time constants but also by the balance of the concentration among transient species^23^.

Figure 2(a) shows the possible energy diagram **S**, **PP** and **P** in P3HT. **S_0_** is the ground state. Plausible transition processes are indicated by arrows. The *k*_i→j_ shown in the Figure denotes the rate constant for the transition process from *i* to *j* states. Deactivation processes from **S**, **PP** and **P** are assumed to be either monomolecular or bimolecular. The *k*_i+i→S0_ is the rate constant for the geminate recombination from *i* state to **S_0_**, which corresponds to the bimolecular deactivation process.

Time dependence of a concentration of transient species can be expressed by a differential equation. In a differential equation for target species, the decrement and increment terms corresponding to the transitions from target species to another form and vice versa, respectively. The coupled differential equations of **S**, **PP** and **P** are listed below, 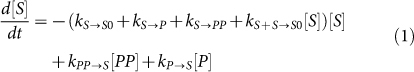

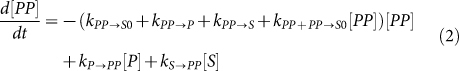

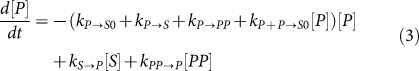
where [*S*], [*PP*], [*P*] are the concentration of **S**, **PP** and **P**, respectively, and are associated with Δ*OD*(*t*) using absorption cross section (*σ*) of each component where *σ* of **S**, **PP** and **P** were assumed to be 2 × 10^−17^ cm^2^ based on the previously published reports^24^,^25^. The detail of this analysis is also described in the supplementary information.

Figure 2(b) shows typical fitting results of Δ*OD*(*t*) contributed from **S**, **PP** and **P** using coupled differential equations at a certain temperature. Open circles and solid lines denote the experimental data and the calculated Δ*OD*(*t*), respectively. Since the calculated Δ*OD*(*t*)s reproduce the experimental data well, it seems most likely that our analysis using differential equations describes the photodynamics of P3HT. Fitting results at different temperatures are shown in Figure S3. The rate constants for the transition processes in P3HT which gave the best-fit Δ*OD*(*t*) curves were plotted as a function of temperature in Figures S4~S6. If the *k* value is nearly equal to zero, the corresponding transition process should not occur. This then follows that the transition processes of **S**→**S_0_**, **S**→**PP**, **S**→**P**, **PP**→**S_0_**, **PP**→**S**, **P**→**S_0_**, **P**→**S** and **P+P**→**S_0_** do not exist under our current experimental condition (Figures S4~S6). Here, the deactivation processes of **P**, that is, **P**→**S_0_** and **P+P**→**S_0_**, were not observed because of the short time scale in our measurement. It should be noteworthy to emphasize here that the **S**→**P** process was not observed in our analysis. This is in contrast to the conclusion obtained from the simple comparison with the decay time constant for **S** and the rise time constant for **P**^17^,^26^.

Therefore, only four processes of **S**+**S**→**S_0_**, **PP**+**PP**→**S_0_**, **PP**→**P** and **P**→**PP** exist. Here we focus on **PP**→**P**. The remaining three processes are discussed in the supplementary information (Figure S4~S6). Figure 2(c) shows the temperature dependence of *k***_PP_**_→**P**_. The *k***_PP_**_→**P**_ values remained constant at 0.10 ps^−1^ below 300 K and then increased with increasing temperature. This means that the dissociation of **PP** to **P** became faster above 300 K.

Since **P** was generated only from **PP** as explained above, the conversion from **PP** to **P** can be simply defined as the number ratio of **PP** to **P**. Figure 2(d) shows the temperature dependence of the conversion from **PP** to **P**. The conversion increased with increasing temperature above 300 K while the conversion is constant to be (35 ± 5)% below 300 K. Since there are only two transition paths from **PP**, **PP**→**P** and **PP**+**PP**→**S_0_**, an increase in the conversion from **PP** to **P** means the suppression of the geminate recombination of **PP**. Hence, it seems reasonable to conclude that above 300 K, the conversion of **P** from **PP** is activated while the geminate recombination of **PP** is suppressed. The photogeneration yields of **PP** and **P** are discussed in the supplementary information (Figure S7). The temperature dependence of the photocurrent of P3HT is shown in Figure S8 in the supplementary information as well. Both Figures S7 and S8 support our claim that the photodynamics in P3HT changed at 300 K.

Steady-state absorption spectra provide information on the conjugation length for P3HT. Figure 3 shows the temperature dependence of the peak maximum wavelength in the steady-state absorption spectra for P3HT in a film. The wavelength became smaller with increasing temperature. This means that the effective conjugation length decreases in the P3HT film with increasing temperature. The decrement of wavelength with temperature changed again at 300 K. This motivates us to study directly the molecular motion of P3HT in the film state.

DMA enables us to gain direct access to the thermal molecular motion of polymers in a film. Once a mode of molecular motion is activated with increasing temperature, a peak in the loss modulus (*E*″) can be observed. Figure 4(a) shows a typical temperature dependence of *E*″ for a P3HT film. Three relaxation peaks were observed at approximately 200, 300 and 390 K, the so-called *β*, *α*_1_, and *α*_2_ processes, at a frequency (*f*) of 20 Hz. Based on the relation between the frequency and the inverse of the temperature at which a peak is observed, the apparent activation energy (ΔH*) for the relaxation process can be extracted as follows; 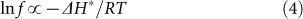
where *R* is the gas constant. Figure 4(b) shows the Arrhenius plots for the three relaxation processes. The ΔH* values for the *β*, *α*_1_, and *α*_2_ processes were 80.6 ± 7.0, 98.7 ± 9.9, and 189.6 ± 23.7 kJ·mol^−1^, respectively. Based on the magnitude of the activation energy for the relaxation process with a complementary structural analysis using Fourier-transform infrared spectroscopy (FT-IR), the *β*, *α*_1_, and *α*_2_ relaxation processes can be assigned to the side chain motion, the twisting motion and the deformation of the interlamellar crystalline region, respectively. Although the details of the analysis are described in the supplementary information (Figure S9), it should be emphasized that these processes are definitely allowed to be released even in the solid state. Thus, it is conceivable that the photodynamics of P3HT is correlated with the twisting motion between coplanar thiophene rings.

We here discuss the relation between the **P** formation and molecular motion. Figure 5 shows a schematic model describing the movement of **PP** in the P3HT film. Below 300 K, π electrons are delocalized along a P3HT chain so that **PP** can move along the chain. This may lead to the geminate recombination of **PP** to **S_0_** due to an increase in the chance for two **PP**s to contact each other. Once the temperature goes beyond 300 K, the twisting motion of thiophene rings is released, resulting in a reduction of the conjugation length of P3HT. This may isolate **PP** in a limited conjugation length and suppress the geminate recombination of **PP**. As a result, the conversion of **PP** to **P** increases. As the temperature, or thermal energy, further increases, the **P** formation from **PP** is also activated because the probability to overcome the potential barrier from **PP** to **P** increases. To confirm our hypothesis mentioned above, sets of similar experiments were made using poly(3-alkyl thiophene)s with various alkyl lengths. Results from the measurements support our claim that the photodynamics changed once the twisting motion of thiophene rings is released.

In conclusion, we have provided the first demonstration of how the photodynamics of poly(thiophene)s in a film is affected not by the crystalline structure, but by the thermal molecular motion of the polymer. Femtosecond transient absorption spectroscopy revealed that **P** was predominantly formed from **PP** at the rate constant of 0.10 ps^−1^ after the photoexcitation, respectively. The **P** formation process from **PP** depended on the temperature. The formation of **P** from **PP** became noticeable above 300 K which was consistent with the *α*_1_ relaxation temperature, at which the twisting motion of thiophene rings was released. The photoelectric conductivity also changed around 300 K. These results clearly indicate that the release of the twisting motion among coplanar thiophene rings regulates the photogeneration process of excitons, as well as the carrier formation dynamics in the P3HT film. This work provides fundamental knowledge for the molecular design of conjugated polymers.

## Methods

### Sample preparation

Regioregular P3HT, poly(3-butylthiophene) (P3BT) and poly(3-octylthiophene)(P3OT) purchased from Sigma-Alidrich Inc. were used as received. Number-average molecular weights (*M*_n_) and polydispersity indices (PDI) of P3HT, P3BT and P3OT were 26, 22 and 58 kg·mol^−1^ and 2.4, 3.1 and 1.6 respectively, which were determined by gel permeation chromatography using polystyrene standards. The melting temperatures of P3HT, P3BT and P3OT measured by differential scanning calorimetry were 488, 500 and 427 K, respectively. Films of polythiophenes were prepared by spin-coating from chloroform solutions onto quartz, barium fluoride (BaF_2_) and commercially available polyimide (PI) substrates for absorption spectroscopy, FT-IR measurements and dynamic mechanical analysis, respectively. The P3HT, P3BT and P3OT films were dried under vacuum at 373, 333 and 310 K for 12 h. The film thickness was approximately 300 nm.

### Steady-state and transient absorption spectroscopy

Steady-state UV-vis absorption spectra were measured with a UV-vis spectrophotometer (Hitachi, U-3500) equipped with a cryostat (Oxford Instruments, Optistat CF-V) at various temperatures from 120 to 420 K. Femtosecond transient absorption spectroscopy were carried out using a pump and probe system. This system consists of a transient absorption spectrometer (Ultrafast Systems, Helios) and a regenerative amplified Ti:sapphire laser (Spectra-Physics, Solstice). The amplified Ti:sapphire laser provided 800 nm fundamental pulses at a repetition rate of 1 kHz with a pulse width of 100 fs (fwhm), which were split into two beams with a beam splitter to generate pump and probe pulses. One beam was converted into pump pulses at 400 nm with a second harmonic generator. The other beam was converted into white light pulses employed as probe pulses in the wavelength region from 400 to 1600 nm. The pump pulses were modulated mechanically with a repetition rate of 500 Hz. The film was set in the cryostat and excited by the pump pulses with 200 *μ*W of laser power. The transient absorption spectra and decays were followed over the time range from −5 ps to 100 ps as a function of time at various temperatures from 120 to 420 K under vacuum.

### DMA measurements

Thermal molecular motion of the P3HT film was examined by DMA using a dynamic viscoelastometer (Rheovibron DDV-01FP, A&D Co., Ltd.). Sinusoidal strain was imposed to a sample with a width of 3 mm and a length of 30 mm within the linear response regime. Applying Takayanagi's parallel model, the loss modulus was extracted from just the P3HT film. The measurements were carried out at a heating rate of 1 K min^−1^ under a dry nitrogen atmosphere.

## Author Contributions

K.T. designed all the experiments and directed the work to completion. D.K. provided the intellectual input for the analyses of the transient absorption spectra. Y.O. performed the experiments and analyses. All the authors contributed to the writing of the manuscript.

## Supplementary Material

Supplementary Information Supplementary information

## Figures and Tables

**Figure 1:**
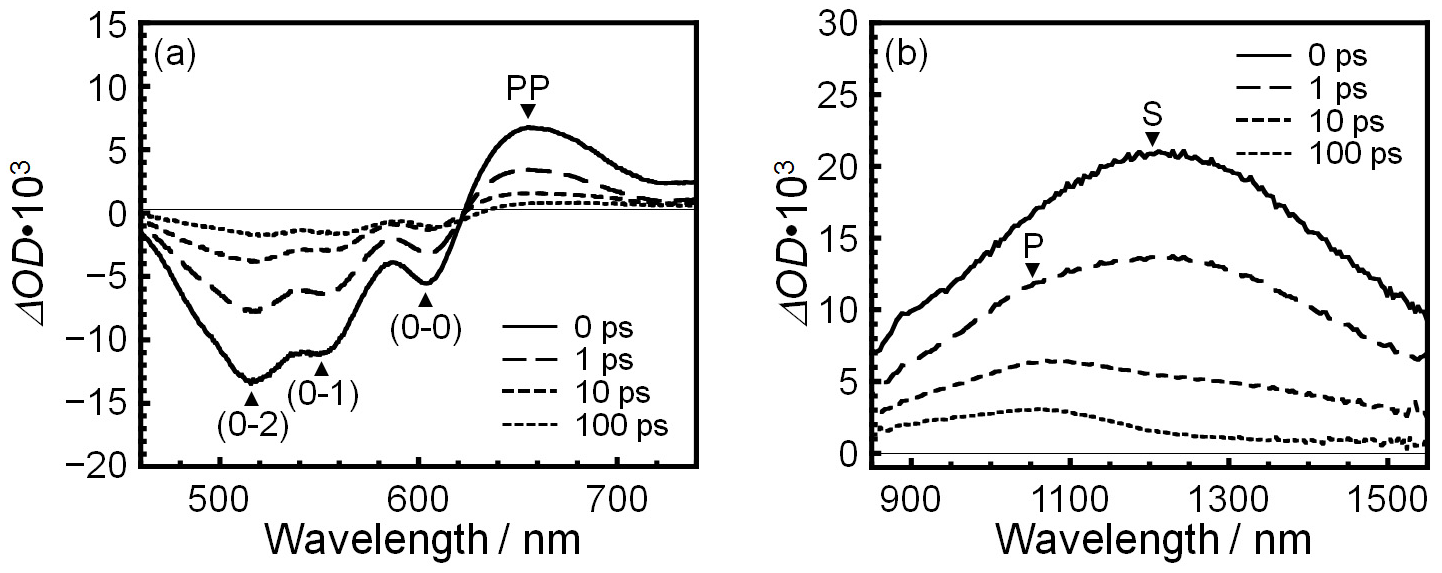
Femtosecond transient absorption of P3HT in a film. Femtosecond transient absorption spectra of a P3HT film at 300 K as a function of delay time in (a) visible and (b) near infrared regions, respectively. Transient absorption signals of P3HT at 1200 nm, 650 nm and 1050 nm correspond to the singlet excitons (**S**), polaron pairs (**PP**) and polarons (**P**), respectively.

**Figure 2:**
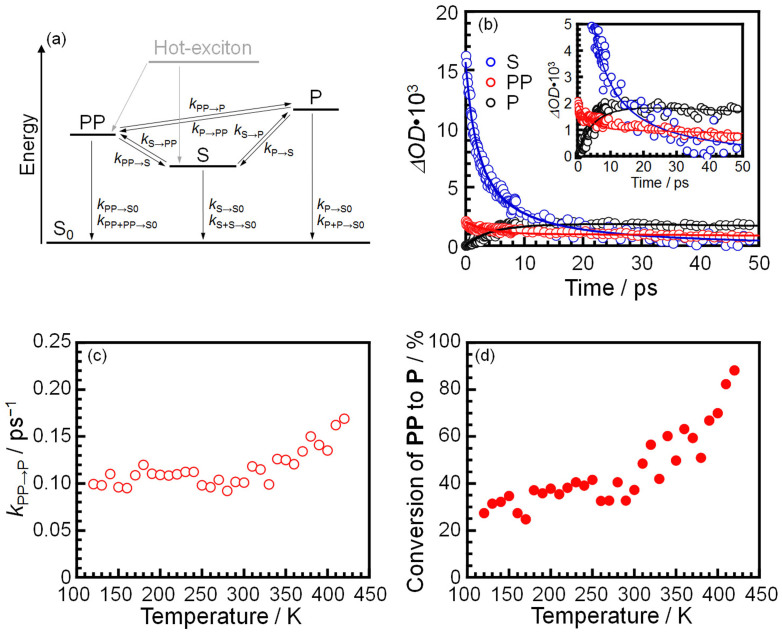
Kinetic analysis of transient species in a P3HT film after photoexcitation. (a) Possible energy diagram of P3HT. (b) Time dependence of optical densities (Δ*OD*(*t*)) for **S**, **PP** and **P** at 420 K. Symbols and solid lines denote experimental data and the fitting results, respectively. (c) Rate constants (*k*) for the **P** formation from **PP**, (d) Temperature dependence of conversion from **PP** to **P**.

**Figure 3:**
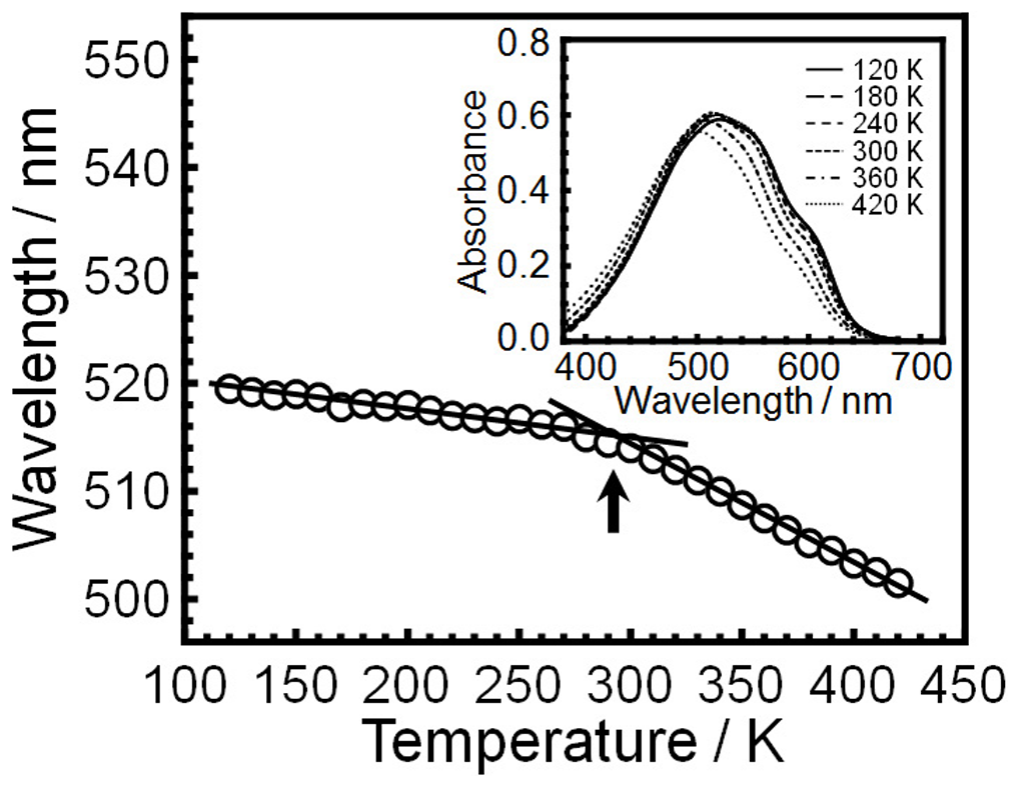
Electronic state of P3HT in a film as a function of temperature. The main panel and inset of this Figure show peak-maximum wavelength in steady-state UV-vis absorption spectra and steady-state spectra at various temperatures, respectively.

**Figure 4:**
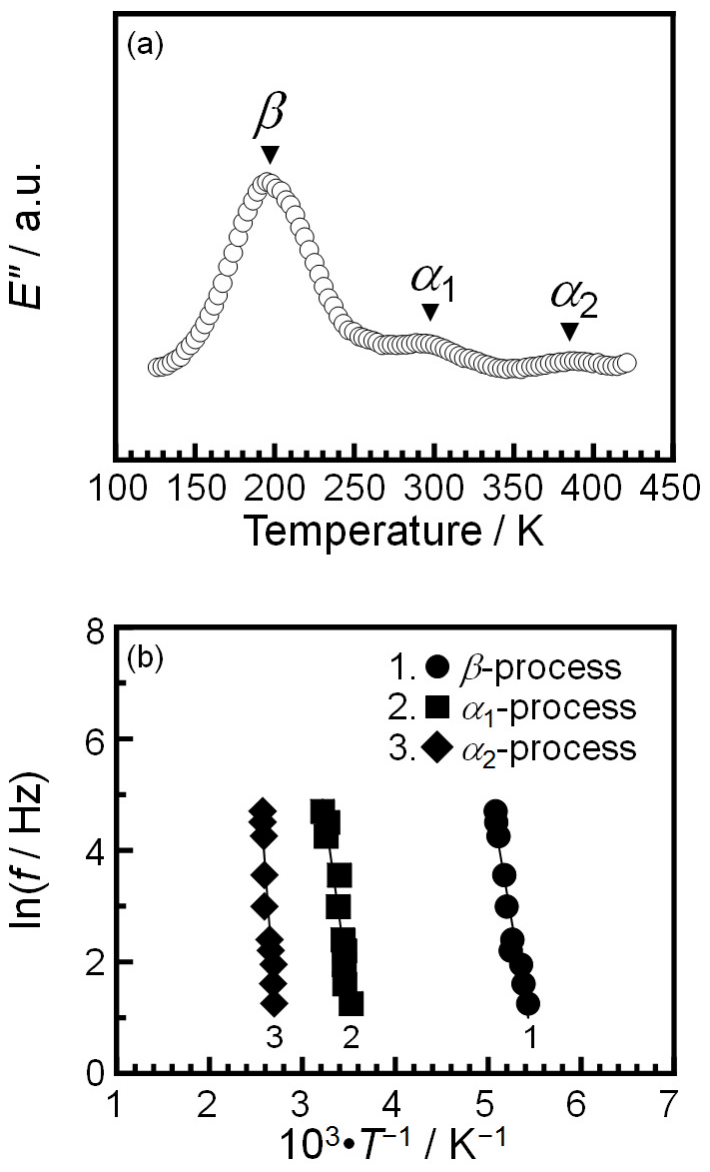
Molecular motion of P3HT in a film as a function of temperature. (a) Dynamic loss modulus (*E*″) for a P3HT film at a frequency of 20 Hz. (b) The relationships between ln *f* and reciprocal absolute temperature for three relaxation processes observed in a P3HT film.

**Figure 5:**
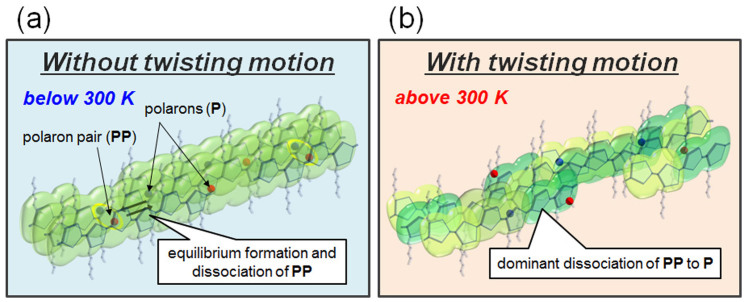
Schematic representation for P formation process from PP in a P3HT film. Isolated red and blue spheres represent **P** with opposite charges and a pair of them is **PP**. (a) Below 300 K, *π* electrons are delocalized along a P3HT chain so that **PP** can move along the chain and geminate recombination of **PP** easily occurs. (b) Above 300 K, the twisting motion of thiophene rings is released. **PP** is isolated in a limited conjugation length and its lifetime becomes longer due to less opportunity for the geminate recombination.
