# Supplementary material for: An Effect of Molecular Motion on Carrier Formation in a Poly(3-hexylthiophene) Film
Source: Sci Rep. 2015 Feb 13;5:8436. doi: 10.1038/srep08436 (PMC4327410; doi:10.1038/srep08436)
Supplement: Supplementary Information [file srep08436-s1.pdf]

Supplementary Information

An Effect of Molecular Motion on Carrier Formation in a Poly(3-hexylthiophene) Film

Yudai Ogata[1], Daisuke Kawaguchi[2*], and Keiji Tanaka[1,3*]

[1]Department of Applied Chemistry, [2]Education Center for Global Leaders in Molecular Systems for Devices and [3]International Institute for Carbon-Neutral Energy Research (WPI-I2CNER), Kyushu University, Fukuoka 819-0395, Japan

Email: d-kawaguchi@cstf.kyushu-u.ac.jp and k-tanaka@cstf.kyushu-u.ac.jp

Tel: +81-92-802-2878

Fax: +81-92-802-2880

Curve fitting analysis of transient absorption spectra for P3HT

Curve fitting was applied to a broad transient absorption peak for P3HT in the near infrared (NIR) region so that it can be resolved into two contributions: from polarons (**P**) and singlet excitons (**S**). Figure S1 shows a transient absorption spectrum of P3HT in a film in the NIR region. Open circles denote the experimental data. Assuming that the experimental data is composed of the sum of two Lorentzian functions corresponding to **P** and **S** with the wavelength maxima of 1050 and 1200 nm, respectively, the peak height and width were changed as variables during the fitting process. The red curve in Figure S1 is the best-fit to the experimental data and is equal to the sum of the contributions from **P** and **S** shown by black and blue curves. This curve fitting analysis was successfully applied at all the temperatures and times. Figure S2 shows typical fitting results at different temperatures and times.

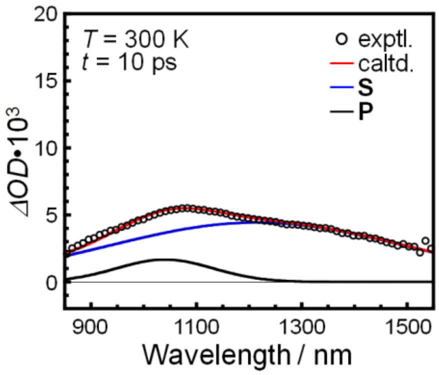

Figure S1. An example of curve-fitting analysis of femtosecond transient absorption spectrum for P3HT in a film in the near infrared region. Open circles denote the experimental data and the red curve is calculated as is the sum of the extracted spectra for **S** and **P**.

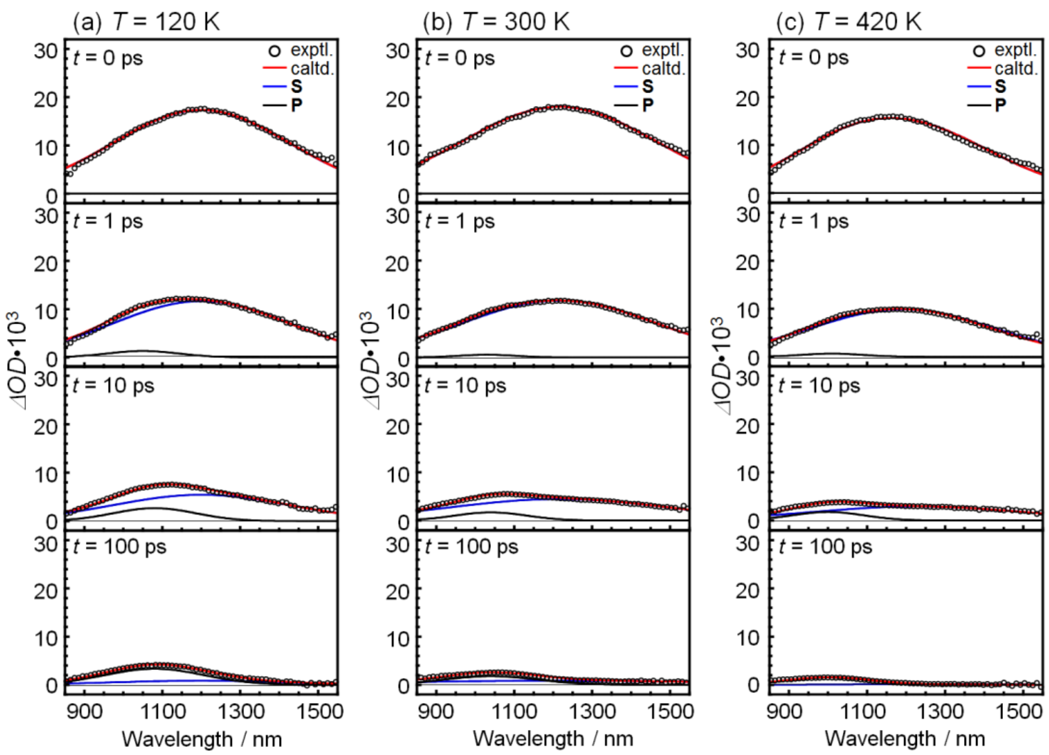

Figure S2. Time evolution of transient absorption spectra for P3HT in the NIR region at (a) 120 K, (b) 300 K and (c) 420 K.

Kinetic analysis for S, PP and P

We analyzed the data with coupled differential equations for the time dependence of concentrations of **S**, **PP** and **P** taking all possible relations among them into account. The deactivation process from **S**, **PP** and **P** is assumed to be either monomolecular or bimolecular processes. The coupled differential equations used in our analyses are as follows,

$$\frac{d[S]}{dt} = -\left(k_{S\to S0} + k_{S\to P} + k_{S\to PP} + k_{S+S\to S0}[S]\right)[S]$$

$$+ k_{PP\to S}[PP] + k_{P\to S}[P] \quad (1)$$

$$\frac{d[PP]}{dt} = -\left(k_{PP\to S0} + k_{PP\to P} + k_{PP\to S} + k_{PP+PP\to S0}[PP]\right)[PP]$$

$$+k_{P\to PP}[P]+k_{S\to PP}[S] \quad (2)$$

$$\frac{d[P]}{dt}=-(k_{P\to S0}+k_{P\to S}+k_{P\to PP}+k_{P+P\to S0}[P])[P]$$

$$+k_{S\to P}[S]+k_{PP\to P}[PP] \quad (3)$$

where [S], [PP], [P] are concentration of **S**, **PP** and **P**, respectively. $\mathbf{S_0}$ denotes the ground state. The $k_{i\to j}$ shown denotes the rate constant for the transition process from *i* to *j* states. The $k_{i+i\to S0}$ is the one for the geminate recombination from an *i* state to $\mathbf{S_0}$. Since the geminate recombination occurs by encountering two excited species, the corresponding concentration change should be expressed as $-k_{i+i\to j}[M]^2$, where M is an excited species.

In this study, [S], [PP], [P] are estimated by the following equations using $\Delta OD_i(t)$ and absorption cross section (σ_i) for an *i* component.

$$[S]=\Delta OD_S(t)/\sigma_S \quad (4)$$

$$[PP]=\Delta OD_{PP}(t)/\sigma_{PP} \quad (5)$$

$$[P]=\Delta OD_P(t)/\sigma_P \quad (6)$$

Here, σ_S, σ_{PP} and σ_P are assumed to be 2×10^{-17} cm^2 based on the previous reports.[1, 2] Thus,

$$\sigma=\sigma_S=\sigma_{PP}=\sigma_P \quad (7)$$

We obtain eqs. (8)~(10).

$$\frac{d(\Delta OD_S)}{dt}=-\left(k_{S\to S0}+k_{S\to P}+k_{S\to PP}+\frac{k_{S+S\to S0}}{\sigma}(\Delta OD_S)\right)(\Delta OD_S)$$

$$+k_{PP\to S}(\Delta OD_{PP})+k_{P\to S}(\Delta OD_P) \quad (8)$$

$$\frac{d(\Delta OD_{PP})}{dt} = -\left(k_{PP\to S0} + k_{PP\to P} + k_{PP\to S} + \frac{k_{PP+PP\to S0}}{\sigma}(\Delta OD_{PP})\right)(\Delta OD_{PP})$$

$$+ k_{P\to PP}(\Delta OD_P) + k_{S\to PP}(\Delta OD_S) \quad (9)$$

$$\frac{d(\Delta OD_P)}{dt} = -\left(k_{P\to S0} + k_{P\to S} + k_{P\to PP} + \frac{k_{P+P\to S0}}{\sigma}(\Delta OD_P)\right)(\Delta OD_P)$$

$$+ k_{S\to P}(\Delta OD_S) + k_{PP\to P}(\Delta OD_{PP}) \quad (10)$$

The time dependence of ΔOD for **S**, **PP** and **P** was fitted using eqs (8)~(10) with k as fitting parameters. Based on the magnitude of k value, it can be judged whether or not a certain transition process as described above exists.

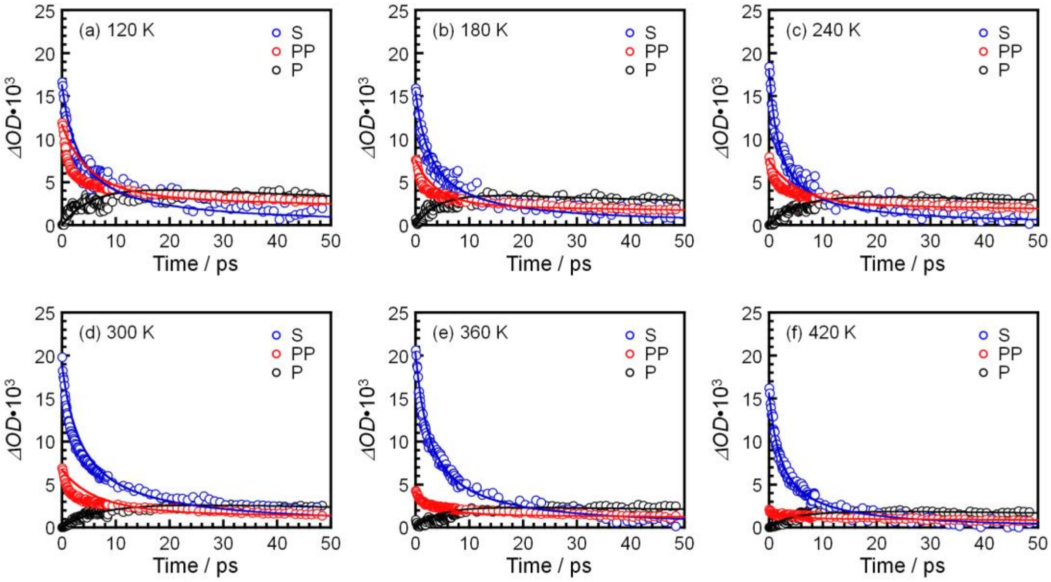

Figure S3. Time dependence of the Δ*OD*(*t*) for S, PP and P at (a) 120 K (b) 180 K (c) 240 K (d) 300 K (e) 360 K and (f) 420 K, respectively. Open circles are experimental data and solid lines denote the best-fit curves obtained, respectively.

S decay process

Figure S4 shows the temperature dependence of k related with the **S** decay process. In this case, the $k_{S\to S0}$, $k_{S\to P}$ and $k_{S\to PP}$ values were 0 at all temperatures. This means that

the **S→S$_0$**, **S→P**, and **S→PP** processes do not exist under the current experimental condition. On the other hand, the $k_{S+S\rightarrow S0}$ values were ca. 4.0×10^{-16} cm^3•ps^{-1} below 300 K and slightly increased with increasing temperature above 300 K, implying that the geminate recombination of **S** occurred and became faster above 300 K.

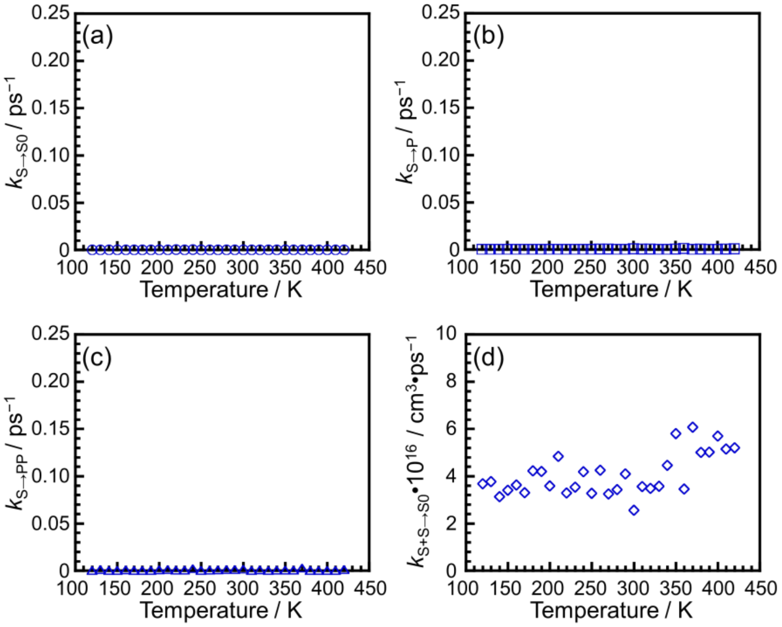

Figure S4. Temperature dependence of rate constant for (a) S to S$_0$, (b) S to P, (c) S to PP and (d) S+S to S$_0$ (geminate recombination), respectively.

PP decay process

Figure S5 shows the temperature dependence of *k* for the **PP** decay process. The **PP→S$_0$** and **PP→S** processes do not exist at all temperatures. In contrast, the **PP→P** and **PP + PP → S$_0$** processes exist and depend on the temperature. Both $k_{\mathbf{PP\rightarrow P}}$ and $k_{\mathbf{PP+PP\rightarrow S0}}$ values were constant to be 0.10 ps^{-1} and 1.5×10^{-16} cm^3•ps^{-1}, respectively, below 300 K and then increased with increasing temperature. This means that both dissociation and geminate recombination processes became faster above 300 K.

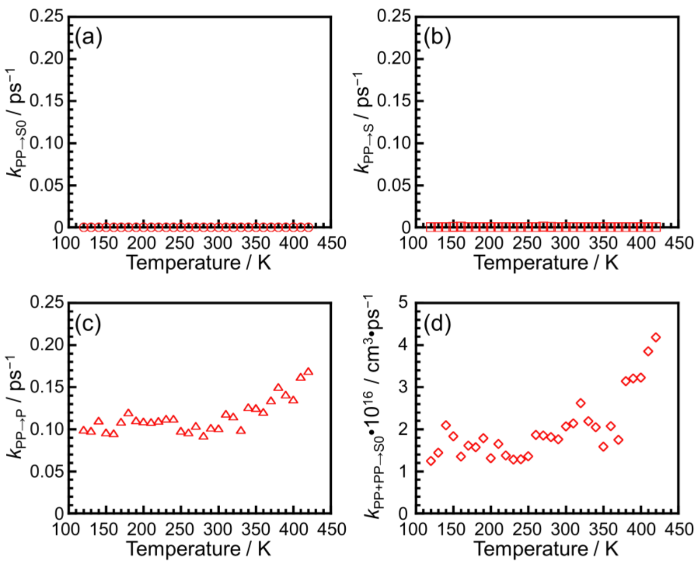

Figure S5. Temperature dependence of rate constant for various processes from PP. (a) PP to S_0, (b) PP to S, (c) PP to P and (d) PP+PP to S_0 (geminate recombination).

P formation process

Figure S6 shows the temperature dependence of *k* for the transitions from **P** to other states. Similarly, the **P→S_0**, **P→S** and **P+P→S_0** processes did not occur under this condition. Both deactivation processes (**P→S_0** and **P+P→S_0**) apparently did not exist due to the lifetime of **P** being longer than the experimental time (~100 ps). The $k_{P\rightarrow PP}$ values remained constant at 0.1 ps^{-1} regardless of the temperature. This value is similar to the $k_{PP\rightarrow P}$ below 300 K, indicating that the formation and the dissociation processes of **PP** are in an equilibrium state. Above 300 K, the $k_{PP\rightarrow P}$ values increased with temperature while $k_{P\rightarrow PP}$ were constant, meaning that the dissociation of **PP** became dominant under the condition.

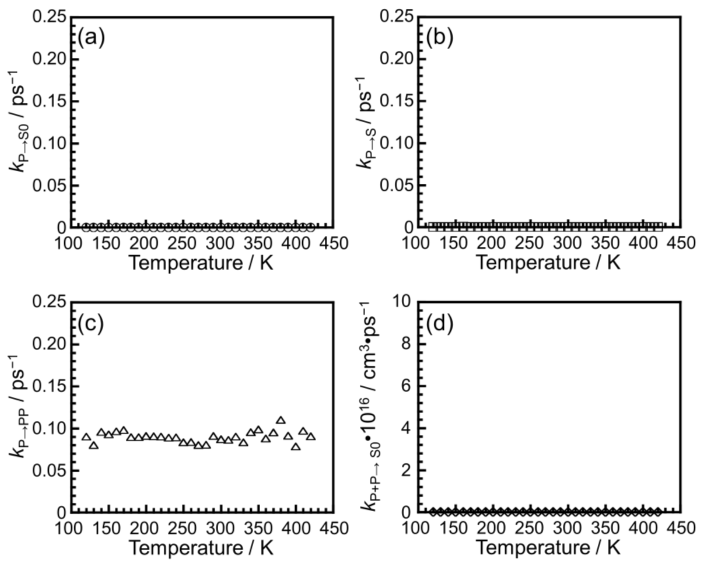

Figure S6. Temperature dependence of the rate constant for (a) P to S_0, (b) P to S, (c) P to PP and (d) P+P to S_0 (geminate recombination), respectively.

Photogeneration yield of PP and P

Figure S7 shows the temperature dependence of the photogeneration yield of **PP** and **P**, which are defined as the number ratio of **PP** or **P** to photons for the incident laser pulse. The photogeneration yield of **PP** was constant at (3.5±0.5) % below 300 K and decreased with increasing temperature above 300 K. The photogeneration of **P** remained the same at (1.2±0.1)% below 300 K but decreased with increasing temperature after passing over 300 K.

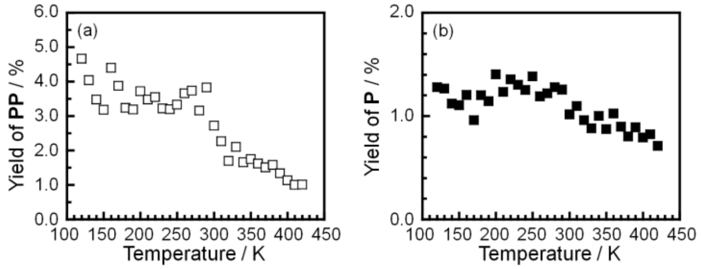

Figure S7. Temperature dependence of photogeneration yield of (a) PP and (b) P.

Temperature dependence of FT-IR spectra for P3HT

Fourier-transform infrared (FT-IR) measurements were carried out to examine the structural changes of P3HT in a film at various temperatures between the α_1 and α_2 relaxation temperatures. Measurements were carried out using a spectrometer (FT-IR620, JASCO International Co., Ltd.) equipped with a cryostat under vacuum. All spectra were recorded from 120 to 420 K at a resolution of 2 cm^{-1} and with an averaging of 64 scans.

Figure S8(a) shows the FT-IR spectra for P3HT in the C_β-H out-of-plane deformation region as a function of temperature. An absorption peak around 820 cm^{-1} and a small shoulder around 835 cm^{-1} were observed. The C_β-H out-of-plane deformation band is sensitive to the crystalline state[3, 4]. Yazawa *et al*. found that the characteristic bands at 820 and 835 cm^{-1} can be assigned to the crystalline and the amorphous states, which correspond to untwisting and twisting motions of thiophene rings, respectively[3].

Figure S8(b) shows the temperature dependence of the areal fraction for the peak at 820 cm^{-1}, indicating the fractional amount of untwisted thiophene rings. The areal fraction was obtained by curve-fitting with two Gaussian peaks with fixed bandwidths at 820 and 835 cm^{-1}, indicating the untwisting and twisting thiophene rings, respectively. The fraction for the 820 cm^{-1} band was constant from 120 to 300 K, and then, started to decrease around 300 K. Also, the slope on the figure became sharper above 390 K. This means that the number of twisted thiophene rings starts to increase and to further do so around 300 and 390 K, respectively. Thus, it is evident that the α_1 and α_2 relaxation processes for P3HT correspond to the twisting motion of thiophene rings in the crystalline region and the deformation of the interlamellar crystalline region, respectively.

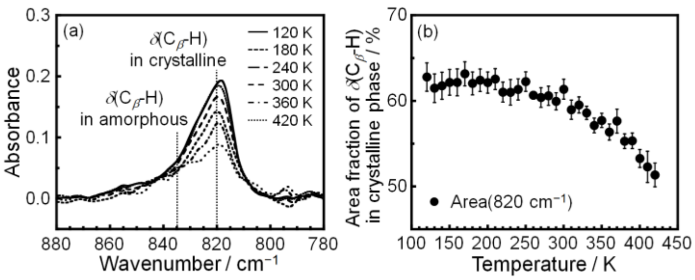

Figure S8. **FT-IR measurements.** (**a**) FT-IR spectra for a P3HT film in the region of 780-880 cm^{-1} (C_β-H out-of-plane deformation band) as a function of temperature. (**b**) Temperature dependence of fractional amount of peak area for 820 cm^{-1} band, indicating the fraction of untwisting thiophene rings.

Temperature dependence of photoelectric conductivity in a P3HT film

The photoconductivity of a P3HT film was examined on the basis of two-point probe resistivity measurements. The resistivity measurements were performed using a Keithley SMU Source Measure Unit at temperatures ranging from 268 to 333 K under vacuum. The film was irradiated with an AM1.5G simulated by white light from a solar simulator (Asahi Spectra, HAL-320) at 100 mW·cm^{-2}.

Figure S9 shows the temperature dependence of the photoelectric conductivity (σ_c) for P3HT in a film examined by a two-point probe resistivity measurement. The σ_c was constant at 0.12 mS·m^{-1} below 300 K and then started to increase from 300 K. According to the variable-range hopping theory, the temperature dependence of σ_c can be expressed as,

$$\sigma_c \propto \exp(-\Delta E/RT) \quad (11)$$

where ΔE is the apparent hopping activation energy. The solid line in Figure S9 is the best-fit curve for the σ_c in a P3HT film using eq. (11) above 300 K. The ΔE value giving the best-fit is 10.5±0.9 kJ·mol^{-1}, being comparable to the reported value for P3HT and

PPV. On the other hand, the dotted line in Figure S9, which is the extrapolated to below 300 K based on the eq. (11) was inconsistent with the experimental data, meaning that the carrier transport does not follow the hopping process in the temperature region lower than 300 K. Hence, it can be concluded that the carrier transport proceeds via the hopping mechanism of **P** in the P3HT film above 300 K due to the activation of the twisting motion for thiophene rings. This is in good accord with our knowledge that the conductivity in a film of poly(alkyl thiophene), or alkyl thiophene, generally follows the hopping mechanism.

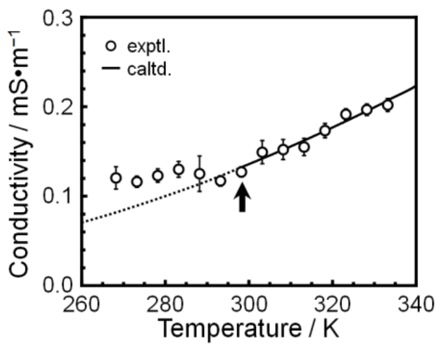

Figure S9. **Temperature dependence of photoelectric conductivity in a P3HT film.** Open circles and a solid line denote experimental data and the best-fit curve, respectively.

References

1. Sheng C. X., Tong M., Singh S., Vardeny Z. V. Experimental determination of the charge/neutral branching ratio eta in the photoexcitation of pi-conjugated polymers by broadband ultrafast spectroscopy. *Physical Review B*, **75** 085206 (2007).

2. Cook S., Furube A., Katoh R. Analysis of the excited states of regioregular polythiophene P3HT. *Energy & Environmental Science*, **1** 294-299 (2008).

3. Yazawa K., Inoue Y., Yamamoto T., Asakawa N. Twist glass transition in regioregulated poly(3-alkylthiophene). *Physical Review B*, **74** 094204 (2006).

4. Yuan Y., *et al.* Polymorphism and structural transition around 54 degrees C in regioregular poly(3-hexylthiophene) with high crystallinity as revealed by infrared spectroscopy. *Macromolecules*, **44** 9341-9350 (2011).
